# Actions of Prolactin in the Brain: From Physiological Adaptations to Stress and Neurogenesis to Psychopathology

**DOI:** 10.3389/fendo.2016.00025

**Published:** 2016-03-30

**Authors:** Luz Torner

**Affiliations:** ^1^Neuroendocrinología, Departamento de Neurociencias, Centro de Investigación Biomédica de Michoacán, Instituto Mexicano del Seguro Social, Morelia, Mexico

**Keywords:** anxiety, depression, prolactin receptors, mood disorders, neurogenesis

## Abstract

Prolactin (PRL) is one of the most versatile hormones known. It is considered an adaptive hormone due to the key roles it plays in the modulation of the stress response and during pregnancy and lactation. Within the brain, PRL acts as a neuropeptide to promote physiological responses related to reproduction, stress adaptation, neurogenesis, and neuroprotection. The action of PRL on the nervous system contributes to the wide array of changes that occur in the female brain during pregnancy and result in the attenuation of the hypothalamic–pituitary–adrenal axis. Together, all these changes promote behavioral and physiological adaptations of the new mother to enable reproductive success. Brain adaptations driven by PRL are also important for the regulation of maternal emotionality and well-being. PRL also affects the male brain during the stress response, but its effects have been less studied. PRL regulates neurogenesis both in the subventricular zone and in the hippocampus. Therefore, alterations in the PRL system due to stress or exposure to substances that reduce neurogenesis or other conditions, could contribute to maladaptive responses and pathological behavioral outcomes. Here, we review the PRL system and the role it plays in the modulation of stress response and emotion regulation. We discuss the effects of PRL on neurogenesis and neuroprotection, the putative neuronal mechanisms underlying these effects, and their contribution to the onset of psychopathological states such as depression.

## Introduction

Prolactin (PRL) is a pleiotropic pituitary hormone with more than 300 known physiological effects. This protein hormone has a regulatory control on reproduction, immunomodulation, angiogenesis, energy metabolism, osmotic balance, and development. In addition to its peripheral functions, PRL also plays many important roles as a neuropeptide. PRL crosses the blood–brain barrier, and local hypothalamic production contributes to reach several brain regions producing strong modulatory effects. In particular, PRL contributes to the regulation of the stress responses through the inhibition of the hypothalamic–pituitary–adrenal (HPA) axis. Moreover, PRL modulates anxiety and depressive-like behaviors. PRL also regulates neurogenesis, the generation of new neurons, in both the subventricular zone (SVZ) and the hippocampus. PRL levels and environmental conditions may induce changes in neurogenesis, potentially leading to an impact on emotional behavior. Indeed, reduction of PRL levels during early pregnancy affects olfactory bulb neurogenesis and increases postpartum anxiety in the female rat. In addition, PRL may exert neuroprotective effects in the hippocampus of adult animals exposed to chronic stress or subjected to hippocampal infusions of kainic acid. However, PRL reduces neurogenesis, when administered, during the early developmental stages and promotes depressive-like behavior in adulthood. Some of these effects are mediated by the activation of different neuronal signaling systems and ion channels. Here, we will summarize some aspects of PRL actions within the brain and their contribution to the onset of psychopathological states like depression.

## The Prolactin System

### PRL Synthesis

Prolactin is produced mainly by the lactotroph cells in the anterior pituitary. PRL is under the inhibitory control by dopamine released from the tuberoinfundibular dopaminergic (TIDA) neurons (1). Despite the abundant efforts to map PRL in cerebral regions using several techniques, the synthesis of PRL within the brain has been brought into question. PRL positive neurons were localized by immunocytochemistry in males and females of different species (2). PRL mRNA was detected by PCR in paraventricular nucleus (PVN) and supraoptic nucleus (3) and by qPCR in the hypothalamus of female rats (4). *In situ* hybridization showed PRL expression in the medial preoptic area (MPOA), periventricular preoptic nucleus, the bed nucleus of the stria terminalis, the PVN, and the lateral septum in the domestic turkey and in both the fetal and adult sheep (5, 6). Recent studies using microarray techniques detected the expression of PRL in the hippocampus of resilient mice (7).

Peripheral PRL is considered the major effector within the brain. It has been hypothesized that PRL may cross the blood–brain barrier through a receptor-mediated mechanism occurring in the cells of the choroid plexus (8). However, recent studies using the PRL receptor (PRL-R) knockout mice showed that PRL transport does not depend on the PRL-R but rather on another, yet to be identified, mechanism (9). Thirty minutes after its peripheral administration, PRL activates brain neurons through the induction of the phosphorylation of Signal Transducer and Activator of Transcription 5 [pSTAT5 (9)]. PRL peaks in the cerebrospinal fluid (CSF) 30 min (10) or 90 min (9) after its peripheral administration. These last studies also suggest that PRL does not access the brain after entering the CSF (9). Since immunoreactive PRL was observed in the choroid plexus and ependymal cells of the cerebral ventricles, it has been hypothesized that PRL may be transported from these sites to reach its target regions (11, 12). However, it is still unknown how PRL accesses its distant target regions within the brain. Whether the ependymal cells, or the neurons, take up PRL and release it in the vicinity of its target cells, or if PRL is released in the extracellular space to diffuse until it reaches its receptors is still a matter of debate.

### PRL Receptors

Prolactin receptors belong to the class 1 cytokine receptor superfamily (1). PRL-R is encoded by a single gene, but alternative splicing produces several PRL-R isoforms. In both humans and rats, three isoforms (long, intermediate, and short – e.g., the size of the intracellular domain) have been identified. The response is triggered after the dimerization of the receptors to form homodimers. Long–long dimers are able to activate second messenger pathways, particularly the JAK-signal transducer and activator of transcription (JAK-STAT) signaling cascade, through the activation of STAT3 and STAT5. The short PRL-R isoform activates the mitogen-activated protein kinase (MAPK) pathway (13). Both long and short receptor heterodimers inhibit PRL signaling (14) and contribute to modulate PRL actions.

## Stress Response and PRL

Stress is a critical factor that may lead to depressive disorders. Stress exposure activates the HPA axis, triggering the release of corticotrophin releasing hormone (CRH) in the PVN, which promotes the secretion of adrenocorticotrophin (ACTH) from the pituitary. In turn, ACTH triggers the release of glucocorticoids from the adrenal glands. PRL is also secreted from the pituitary in response to a number of stressors. Initial studies suggested that PRL may counteract glucocorticoid actions on the immune system during the stress response (15). However, more recent studies showed that preventing PRL-R expression in the brain *via* an antisense probe strongly increases the stress-induced ACTH secretion in virgin and lactating rats, suggesting that PRL plays an inhibitory role on HPA axis reactivity (16, 17). It has been hypothesized that PRL modulates the activity of the HPA axis through a reduction of neural inputs to the PVN. Both acute and chronic PRL intracerebroventricular administrations in virgin female rats reduce neuronal activation in the amygdala and CRH hypothalamic expression in response to stress (18). Additionally, PRL is locally released from the PVN and MPOA in response to physiological stimuli, including stress (4).

Prolactin has been recently associated with resilience in a model of chronic mild stress (CMS). Adult rats, previously subjected to CMS, were selected as responders and non-responders, according to their reaction to stress. The resilient animals (i.e., non-responders) presented higher plasma levels of PRL, and higher PRL-R mRNA in the choroid plexus than their vulnerable (i.e., responders) counterpart (19). Accordingly, a microarray study performed on heterozygous 5-Htt-deficient offspring subjected to prenatal stress showed that PRL, growth hormone, and galanin receptor 3 were differentially expressed in the hippocampus of resilient mice when compared to controls (7). These results suggest a role for PRL in stress regulation at hippocampal level.

## PRL and Emotional Responsiveness

The effects of PRL on anxiety and depressive behavior have been studied, but they differ depending on the species used and the physiological state.

### PRL and Anxiety

Intracerebroventricular or intravenous administrations of PRL exert dose-dependent anxiolytic effects in both male and virgin female rats (16). Anxiety levels in pregnant and lactating rats undergo a progressive reduction (20, 21). These effects are likely to be mediated by PRL, as suggested by the increased anxiety displayed by lactating female rats in the elevated plus maze after blockade of PRL-R brain expression (17). Chronic ICV administration of PRL to ovariectomized E2-replaced female rats (used to simulate the endocrine status during pregnancy) was shown to reduce anxiety levels in the elevated plus maze (18). Taken together, these results strongly suggest that PRL has an anxiolytic effect in rodents.

Studies on inbred lines of rodents have shown an association between elevated levels of PRL and increased anxiety. Increased basal and stress-induced levels of PRL were reported in male rats bred for high anxiety behavior (HAB) as compared to low anxiety behavior (LAB) rats (22). The Roman low-avoidance (RLA/Verh) line of rats (i.e., rats selected for low performance in a two-way active avoidance test) displays increased stress responses, higher levels of PRL, and a passive coping style compared to the Roman high-avoidance (RHA/Verh) line of rats (i.e., rats selected for high performance in a two-way active avoidance test) counterpart (23). The elevated levels of PRL found in this animal line could stem from an imbalance in one or more secretagogues of PRL (serotonin, GABA, etc.). Alternatively, high PRL concentrations could help to improve the excessive reactivity of the HPA axis observed in these animals. In contrast, clinical studies in humans have indicated a correlation between high PRL levels and psychological distress. Female patients with hyperprolactinemia usually report more symptoms of anxiety and hostility than control female subjects (24).

### PRL and Depressive-Like States

Artificially inducing hyperprolactinemia in adult male rats by placing pituitary grafts of a donor rat in the kidney of the receptor animal was shown to exert antidepressive-like effects in the forced swimming test (25).

In humans, some patients with hyperprolactinemia exhibit depressive symptoms. However, no differences were observed in the prevalence of depression in hyperprolactinemic patients compared to controls (24, 26). Remarkably, few studies have made a distinction of the origin of hyperprolactinemia (e.g., a pituitary tumor, or dysregulation of neural pathways due to neurotransmitter alterations) and its relationship with anxiety and depression. Since PRL modulates the expression of its own receptors, it has been hypothesized that hyperprolactinemic patients may express more PRL-R in the brain. However, it is unknown if the receptors are functionally activated. Excessive levels of PRL may prevent the formation of the homodimers necessary for the physiological functions of PRL. Additionally, long-term hyperprolactinemia reduces the ability of the tuberoinfundibular neurons to synthesize dopamine (27). High PRL concentrations generate elevated levels of the 16K PRL fragment called vasoinhibin. This fragment exerts effects opposite to those of the native hormone (28, 29).

### Depressive-Like States and Motherhood

Several animal studies report a causal link between reduced maternal care and depressive-like state or alterations of emotional behavior in the offspring. Stress exposure during pregnancy alters the neural mechanisms that prepare the female to her maternal role and contributes to the development of psychiatric diseases such as depression or anxiety (30, 31). Female rats subjected to chronic psychosocial stress during pregnancy exhibit a postpartum depressive-like state (32–34). The activation of oxytocin and PRL systems during lactation contributes to the attenuation of the HPA axis activation and triggers a positive mood (35). Therefore, alterations in these systems could contribute to the development of the affective disorders observed in the postpartum period. Indeed, oxytocin expression is reduced in the hypothalamus of postpartum female rats subjected to chronic stress during pregnancy (34). Postpartum levels of circulating oxytocin are decreased in women showing depressive symptoms during pregnancy (36). Low concentrations of oxytocin could increase the risk to develop postpartum depression (37).

Dysregulation of PRL-R responsivity could be associated with postpartum disorders (38). Indeed, low levels of PRL were found in women suffering from postpartum depressive disorder (39). Administration of bromocriptine in the early stages of pregnancy reduces PRL levels, induces a depressive-like state, and impairs maternal behavior in female rats, suggesting that PRL may play a key role in the onset of postpartum depression (40, 41). Moreover, breastfeeding-induced raise in PRL levels results in positive effects on both the mother’s health and the mother–infant bonding. A correlation was found between stress, dysphoric moods, and reduced levels of interferon gamma in mothers using bottle feeding (42). On the contrary, breastfeeding mothers scored less in anxiety, depression, and anger tests (43).

## Neurogenesis and Neuroprotection

Chronic stress exposure and depressive states are known to affect neurogenesis. Neurogenesis is the process that produces new neurons throughout life. New neurons are thought to transiently increase neuronal communication. Two neurogenic niches have been recognized in the adult brain: the hippocampus and the SVZ. The hippocampus exerts a negative control on the HPA axis activity and it is involved in emotional modulation. Antidepressive treatments increase hippocampal neurogenesis and show a correlation with mood improvement (44). Olfactory bulb neurogenesis and olfactory dysfunction are also decreased by CMS exposure, a procedure that is known to induce depressive-like states (45).

Prolactin is a regulator of neurogenesis. PRL receptors are expressed in the SVZ and the hippocampus (46–48). Initial evidence of the relationship between PRL and neurogenesis has been reported by studies showing an increase in neurogenesis in the SVZ of pregnant females. This increase was found to be mediated by PRL (47), and it was hypothesized that the olfactory discrimination of odor cues related to pups is critical for maternal success. Other olfactory signals are important cues that also induce neurogenesis in the SVZ and in the hippocampus. Exposure to pheromones from a dominant male induces cell proliferation in both the olfactory bulb and the hippocampus of female mice. These effects are mediated by PRL in the olfactory bulb and luteinizing hormone in the hippocampus, and both hormones contribute to the regulation of female reproduction (49). Exposure to male pheromones increases SVZ neurogenesis and promotes maternal behavior in virgin and postpartum females (50). Injections of bromocriptine, a dopamine agonist, in female rats during the first days of pregnancy to lower PRL levels, reduces olfactory neurogenesis and induces behavioral alterations postpartum (41). These reports clearly suggest that PRL may regulate SVZ neurogenesis and play a key role in mood regulation.

The reduction of hippocampal neurogenesis due to chronic stress exposure was prevented by daily PRL administration in male mice. PRL was shown to promote neuronal fate and to exert neuroprotective actions in the hippocampus (51). PRL effects were initially thought to counteract glucocorticoid reduction of neurosphere proliferation and survival. However, incubation of PRL together with dexamethasone was unable to inhibit dexamethasone effects on neurospheres, despite the induction of ERK1/2. Therefore, it was suggested that PRL may affect hippocampal neurogenesis through an indirect mechanism (52). Following studies showed both *in vitro* (in primary adult hippocampal cells) and *in vivo* (direct injection into the dentate gyrus of adult mice) that PRL administration increases the neurosphere number, suggesting a direct effect of this neuropeptide on the hippocampus (53). Furthermore, these authors reported behavioral deficits in the PRL null mice trained in hippocampus-dependent learning tasks, and suggested a role of PRL in the learning and memory processes (53). In contrast, daily injections of PRL in rats during the first two postnatal weeks reduces neurogenesis in both the dentate gyrus and the olfactory bulb and promotes a depressive-like state in both adult male and female rats (54). These results suggest that PRL exerts different actions on neurogenesis depending on the age of the animals and it could be associated with emotional regulation.

During pregnancy and lactation, PRL protects the hippocampus against the high concentrations of glucocorticoids (55). Administration of ovine PRL to the cerebral ventricle of ovariectomized virgin females buffers the neurodegenerative process induced by kainic acid infusion in CA1, CA3, and CA4 areas of the hippocampus (56, 57). These results suggest a neuroprotective role for PRL in the hippocampus.

## Neural Mechanisms of PRL Actions

Prolactin effects on neurogenesis are mediated by its activation of the extracellular signal-regulated kinase 5 (ERK5). ERK5 is expressed in the neurogenic niches of the brain (58). Moreover, PRL increases both the expression and protein levels of Nestin and microtubule-associated protein 2 (MAP2) in neuroblastoma (SK-N-SH) cells. This suggests that PRL regulates cytoskeletal protein synthesis and therefore contributes to neuronal differentiation (59). Furthermore, PRL modulates Ca^2+^-dependent K^+^ channels, which could affect the release of neurotransmitters in different cerebral regions (60). In the hypothalamus, PRL stimulates the ERK/MAPK pathway in CRH, vasopressin, and oxytocin neurons (61) and promotes EGR-1 expression in magnocellular neurons (62). Activation of these pathways by PRL could contribute to the plastic changes observed in the brain during pregnancy. PRL stimulation of CRH transcription in hypothalamic cultured cells has been interpreted as an indirect inhibitory control of PRL on HPA axis activity (62). However, it is still unknown how PRL affects the HPA axis.

## Concluding Remarks and Future Directions

Prolactin alters neural circuits to help the individual to cope with stress. Reduced activation of neural inputs, activation of ionic channels, or the modulation of several signaling pathways are some of the putative mechanisms of action underlying the effects of PRL on brain circuits. It is unknown how PRL regulates the HPA axis function during the stress response. PRL could affect hypothalamic and/or hippocampal activity to regulate emotionality. The contribution of PRL to the onset of postpartum depression is still unknown. Low PRL levels in nursing women have been associated with postpartum depression. Inhibition of PRL reduces neurogenesis in the olfactory bulb, but the mechanisms that result in a postpartum depressive state are unknown. Besides the pathways affected by PRL to regulate anxiety are not yet known, and whether the PRL effects on hippocampal neurogenesis contribute to modulate anxiety is still uncertain. Administration of PRL in the early postnatal stages reduces neurogenesis in the hippocampus and the olfactory bulb and promotes a depressive-like state in adulthood. This suggests that PRL affects a critical window during brain development and that PRL actions are dependent on the age during which it is administrated. Further studies are needed to understand the mechanisms of action of PRL in the brain, and how this hormone modulates emotionality and anxiety at different developmental stages.

## Author Contributions

The author confirms being the sole contributor of this work and approved it for publication.

## Conflict of Interest Statement

The author declares that the research was conducted in the absence of any commercial or financial relationships that could be considered as a potential conflict of interest.
